# Does atorvastatin therapy change the anti‐Xa activity in xabans‐treated patients with atrial fibrillation?

**DOI:** 10.1002/prp2.730

**Published:** 2021-05-13

**Authors:** Ingrid Škorňová, Matej Samoš, Tomáš Bolek, Lucia Stančiaková, Ľubica Vádelová, Peter Galajda, Ján Staško, Peter Kubisz, Marián Mokáň

**Affiliations:** ^1^ National Centre of Hemostasis and Thrombosis Department of Hematology and Blood Transfusion Jessenius Faculty of Medicine in Martin Comenius University in Bratislava Martin Slovak Republic; ^2^ Department of Internal Medicine I Jessenius Faculty of Medicine in Martin Comenius University in Bratislava Martin Slovak Republic

**Keywords:** apixaban, atorvastatin, atrial fibrillation, rivaroxaban, xabans anti‐Xa activity

## Abstract

Atorvastatin and direct oral factor Xa inhibitors (xabans) are frequently co‐administrated in patients with atrial fibrillation (AF). However, no studies investigating the possibility of the pharmacologic interaction between these agents have been conducted. The aim of this prospective observational study was to determine the impact of atorvastatin therapy on anti‐Xa activity in xabans‐treated patients with AF. We enrolled 115 AF patients on long‐term rivaroxaban (52 patients) and long‐term apixaban (63 patients) therapy. Long‐term atorvastatin (40 mg once daily) was administrated to 28 rivaroxaban‐treated patients and to 28 apixaban‐treated patients. Trough and peak samples were tested for anti‐Xa activity with drug‐specific anti‐Xa chromogenic analysis. For rivaroxaban, there were no significant differences in trough activity (45.5 ± 39.5 ng/ml vs. 46.2 ± 30.1 ng/ml; *p* = .34) and peak anti‐Xa activity (179.2 ± 108.8 ng/ml vs. 208.1 ± 104.1 ng/ml; *p* = .94) between atorvastatin‐treated patients and those without atorvastatin. Similarly, atorvastatin did not impact the trough activity (127.7 ± 71.1 ng/ml vs. 100.8 ± 61.1 ng/ml; *p* = .12) or peak anti‐Xa activity (213.8 ± 103.6 ng/ml vs. 179.3 ± 72.9 ng/ml; *p* = .14) among apixaban‐treated patients with AF. This observational study did not show a significant impact of atorvastatin on trough and peak anti‐Xa activity in xabans‐treated patients with AF.

## Graphical abstract

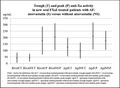

**Abbreviations**
AF: atrial fibrillation
CYP: cytochrome P450
P‐gp: glycoprotein‐P

## 1 INTRODUCTION

Xabans—direct factor Xa inhibitors (Rivaroxaban, Apixaban, and Edoxaban) are orally administrated non‐vitamin K‐dependent direct factor Xa inhibitors (NOACs)^1^, ^2^, ^3^ with few strong drug interactions that are approved for the prevention of embolic events in patients with atrial fibrillation (AF). However, several drug interactions can decrease or increase plasma anti‐Xa activity in direct oral factor Xa inhibitors‐treated patients, and this changed activity could be connected to a relatively higher risk of thrombotic or bleeding events.^4^, ^5^ Statins are relatively frequently co‐administrated with direct oral factor Xa inhibitors due to previous stroke, concomitant ischemic heart or other vascular disease or dyslipidemia in patients with AF. Atorvastatin, the most frequently used statin, is metabolized by cytochrome P450 (CYP) 3A4, and is a substrate of glycoprotein‐P (P‐gp).^6^, ^7^, ^8^ Similarly, xabans are metabolized by CYP 3A4 and CYP2J2, and are substrates of P‐gp^1^, ^2^, ^3^; therefore, there is a theoretical possibility of drug interaction between atorvastatin and direct oral factor Xa inhibitors. Unfortunately, no studies of this possible interaction in patients with AF have been conducted.^9^ Therefore, the aim of this prospective observational study was to determine the impact of atorvastatin therapy on the anti‐Xa activity in direct factor Xa inhibitors‐treated patients with AF.

## 2 PATIENTS AND METHODS

We enrolled consecutive patients with AF on long‐term rivaroxaban or long‐term apixaban therapy requiring hospitalization for symptomatic heart failure or uncontrolled tachycardia (atrial fibrillation with uncontrolled ventricular response) at our Department of Internal Medicine from the beginning of August 2019 to the end of November 2019. All of the patients received long‐term rivaroxaban or apixaban for the prevention of stroke and systemic embolism prior to hospitalization (the decision regarding the agent—rivaroxaban or apixaban was established by their out‐patient cardiology consultant), and continued with this therapy during hospitalization. Patients with a reduced glomerular filtration rate (calculated glomerular filtration rate 15–50 mL/min/1.73 m^2^) were given a lower dose of rivaroxaban (15 mg once daily); a lower dose of apixaban (2.5 mg twice daily) was given to patients who matched two from three of following criteria: reduced glomerular filtration rate (calculated glomerular filtration rate 15–50 mL/min/1.73 m^2^; calculated using “Modification of Diet in Renal Disease—MDRD” formula: estimated glomerular filtration rate in mL/min/1.73 m^2^ = 175 × serum creatinine^−1.154^ × age^−0.203^ × 1.212 (if patient is black) × 0.742 [if female]), age ≥80 years, body weight <60 kg. Long‐term atorvastatin (40 mg once daily) was administered for dyslipidemia, stroke or cerebrovascular disease, and ischemic heart disease. The study design matched the one used in our prior NOACs pharmacology studies.^10^, ^11^, ^12^ We used an observational design. All samples were taken during the in‐hospital stay. We tested the trough samples (24 h after the previous drug dose in rivaroxaban‐treated patients, and 12 h for apixaban‐treated patients; at 7:00 a.m) and peak samples (2 h after the next drug dose administration for rivaroxaban‐treated patients, and 3 h for apixaban‐treated patients; at 9:00 and 10:00 a.m.), used healthcare professional compliance verification, and allowed an attending physician‐based decision on concomitant medication. Similarly, the exclusion criteria, namely a disabling or recent stroke, recent or pending surgery, known inherited bleeding disorders, uncontrolled hypertension, the need for anticoagulation for disorders other than AF, severe renal dysfunction (glomerular filtration rate <15 mL/min/1.73 m^2^), active liver disease and extreme body weight (body mass index <18 kg/m^2^ and >40 kg/m^2^), matched those used in our previous studies. Rivaroxaban therapy lasted on average 110 days prior to sample taking, apixaban therapy lasted 117.5 days, and atorvastatin was taken on average 90.5 days prior to blood sampling, respectively. This study was performed according to all ethical standards defined by the Declaration of Helsinki and approved by the local ethical committee (Jessenius Faculty of Medicine in Martin, Comenius University in Bratislava). The patients agreed to participate in the research. All samples were taken after obtaining a written informed consent to participate in the study. Rivaroxaban and apixaban anti‐Xa activity (ng/ml) was determined using drug‐specific anti‐Xa chromogenic analysis.^13^

### 2.1 Statistical analysis

Data analysis was performed using STATISTICA v 5.0 (StatSoft, Tula, USA). Data were checked for normality with the Shapiro–Wilk test (data are displayed as mean ± standard deviation in case of normally distributed ones and as median and range in case of asymmetrically distributed ones); a *t*‐test was used in the case of normally distributed data or a Mann–Whitney *U* test was used when data distribution was asymmetrical. The *p*‐value of <.05 was considered as a statistically significant difference.

### 2.2 Nomenclature of targets and ligands

Key protein targets and ligands in this article are hyperlinked to corresponding entries in http://www.guidetopharmacology.org, the common portal for data from the IUPHAR/BPS Guide to PHARMACOLOGY,^14^ and are permanently archived in the Concise Guide to PHARMACOLOGY 2019/20.^15^, ^16^

## 3 RESULTS

Between August and November 2019, 115 consecutive patients with AF (41 men, 74 women, average age 74 years, ranging from 65 to 83 years of age) on long‐term rivaroxaban (52 patients; rivaroxaban 15 mg once daily for 32 patients and rivaroxaban 20 mg once daily for 20 patients) or long‐term apixaban therapy (63 patients; apixaban 2.5 mg twice daily for 32 patients and apixaban 5 mg twice daily for 31 patients) were enrolled in our study. Long‐term atorvastatin (40 mg once daily) was administrated to 28 rivaroxaban‐treated patients (8 patients for dyslipidemia, 11 patients for cerebrovascular diseases, and 9 patients for ischemic heart disease) and to 28 apixaban‐treated patients (9 patients for dyslipidemia, 10 patients for cerebrovascular diseases, and 9 patients for ischemic heart disease). There were no significant differences in the length of rivaroxaban and apixaban therapy, concomitant therapy (except of atorvastatin) or basic demographic data (Table 1) between statin‐treated patients and patients without statin therapy. The lipid profile in statin‐treated patients and individuals without statin therapy is reported in Table 1. Ten rivaroxaban‐treated patients in both groups (i.e., 10 patients with atorvastatin and 10 patients without atorvastatin) were treated with 20 mg once daily; and the other patients were treated with 15 mg once daily. Similarly, 13 apixaban‐treated patients in both groups were treated with apixaban 5 mg twice daily, and the other patients were treated with 2.5 mg of apixaban twice daily. No bleeding or ischemic event (stroke or systemic embolism) occurred during the in‐hospital stay.

**TABLE 1:** Basic demographics and medication in studied AF patients on long‐term rivaroxaban/apixaban therapy

Parameter	Rivaroxaban‐treated patients with statin	Rivaroxaban‐treated patients without statin	Significance (*p* value)	Apixaban‐treated patients with statin	Apixaban‐treated patients without statin	Significance (*p* value)
Number of patients (men/women) (absolute number)	28 (9/19)	24 (8/16)	N/A	28 (12/16)	35 (10/25)	N/A
Age (years)	75 (68–81)	72 (65–83)	0.34	75 (69–80)	76 (68–84)	.64
Beta‐blockers (%)	96.4	91.6	0.88	96.4	85.7	.12
Diuretics (%)	78.5	75.0	0.21	64.2	42.8	.51
Amiodarone (%)	3.5	12.5	0.26	10.7	14.2	.64
Verapamil (%)	3.5	8.3	0.65	0	5.7	.16
Digoxin (%)	25.0	37.5	0.34	39.2	37.1	.93
ACE inhibitors, AT1RB (%)	58.5/20.0	33.3/33.3	0.76	60.7/14.2	42.8/11.4	.10
PPI (%)	71.4	45.8	0.23	60.7	62.8	.87
Duration of rivaroxaban/apixaban (days)	117.5	117.5	0.75	110	110	.96
Duration of statin (days)	90.5	0	N/A	85.5	0	N/A
BMI (kg/m^2^)	26.5	27.5	0.57	27.1	27.4	.77
CHA2DS2VASc (score)	4.5	3.9	0.15	5.3	5.1	.42
HAS‐BLED (score)	3.5	3.25	0.72	3.8	3.5	.30
Serum creatinine (umol/L)	99.9 ± 22.9	111.6 ± 28.8	0.13	111.1 ± 46.7	116.8 ± 44.3	.60
Calculated GFR ‐ MDRD (ml/min/1.73 m^2^)	60.2	53.7	0.17	59.0	51.9	.21
Total bilirubin (mmol/L)	12.8 ± 6.6	19.1 ± 14.1	0.08	14.8 ± 8.8	13.8 ± 7.1	.64
Total serum protein (g/L)	64.7 ± 7.5	62.7 ± 7.9	0.40	65.6 ± 9.8	63.5 ± 11.1	.52
Total serum cholesterol (mmol/L)	4.0 ± 1.7	4.6 ± 1.5	0.31	4.2 ± 1.1	4.3 ± 1.1	.84
LDL‐cholesterol (mmol/L)	2.1 ± 1.5	2.8 ± 1.4	0.21	2.4 ± 0.8	2.5 ± 0.9	.72
HDL‐cholesterol (mmol/L)	1.2 ± 0.5	1.1 ± 0.5	0.94	1.2 ± 0.3	1.2 ± 0.4	.97
Triglycerides (mmol/L)	1.4 ± 0.6	1.2 ± 0.5	0.24	1.3 ± 0.6	1.5 ± 0.9	.45
Diabetes mellitus (%)	42.8	33.3	0.49	39.2	34.2	.58
History of stroke (%)	42.5	21.7	0.18	35.7	28.5	.44

Abbreviations: ACE, angiotensin‐converting enzyme; AT1R, AT1 receptor; BMI, body mass index; CHA2DS2VASc, Congestive heart failure or left ventricular dysfunction Hypertension, Age ≥75 (doubled), Diabetes, Stroke (doubled)‐Vascular disease, Age 65–74, Sex category; GFR, glomerular filtration rate; HAS‐BLED, Hypertension, Abnormal liver/renal function, Stroke history, Bleeding history or predisposition, Labile INR, Elderly, Drug/alcohol usage; HDL, high‐density lipoproteins; LDL, ow‐density lipoproteins; MDRD, Modification of Diet in Renal Disease; N/A, non‐applicable; PPI, proton pump inhibition.

**FIGURE 1:**
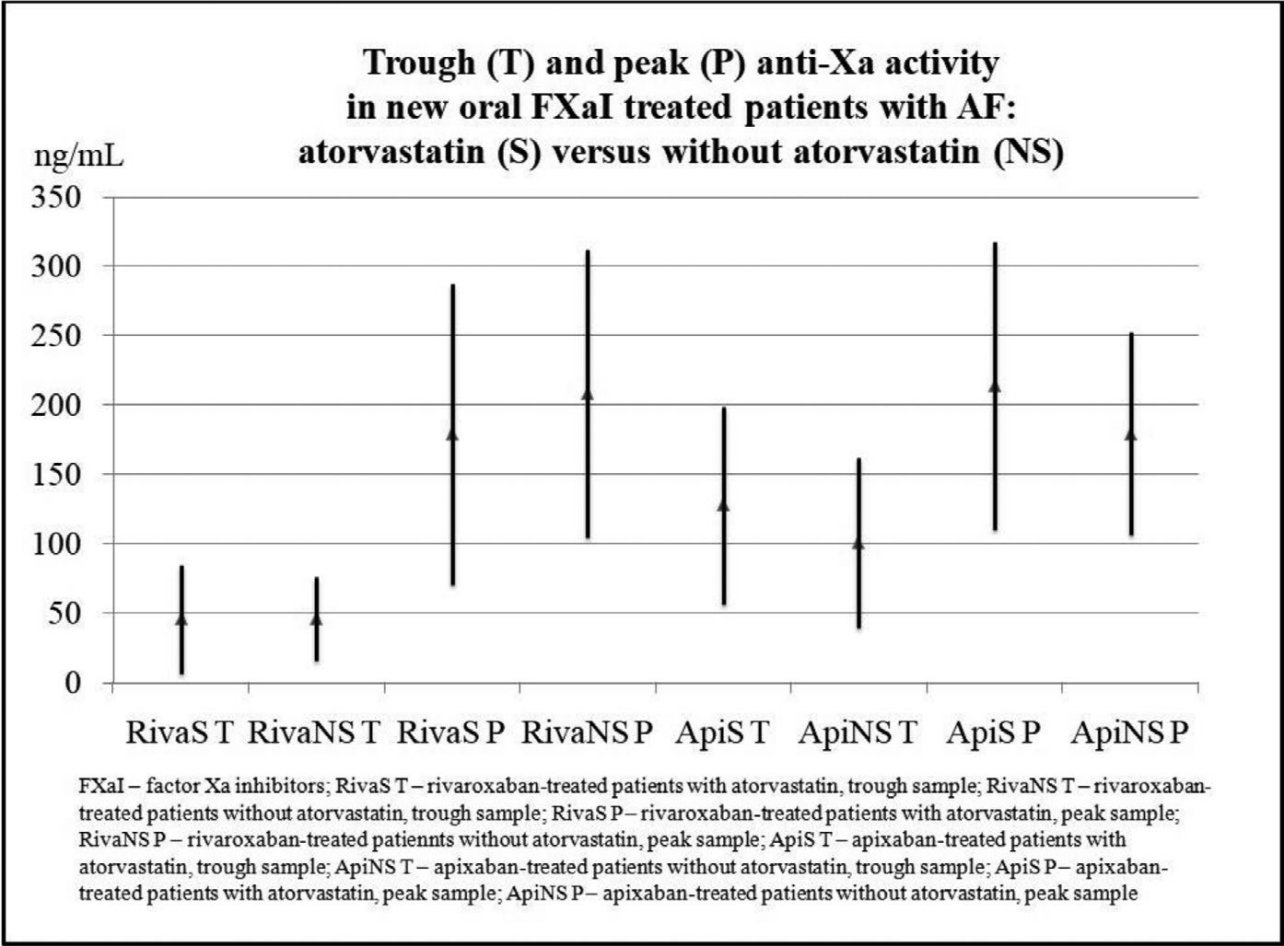
Impact of atorvastatin on trough and peak anti‐Xa activity in AF patients on long‐term rivaroxaban/apixaban therapy

Comparing rivaroxaban‐treated patients on atorvastatin with those without statin therapy (Figure 1), there were no significant differences in trough anti‐Xa activity (45.5 ± 39.5 ng/ml vs. 46.2 ± 30.1 ng/ml; *t*‐test; *p* = .34) and peak anti‐Xa activity (179.2 ± 108.8 ng/ml vs. 208.1 ± 104.1 ng/ml; *t*‐test; *p* = .94). Subsequently, patients with amiodarone and verapamil co‐administration were excluded from the analysis (considering the slight imbalance between patients treated with or without atorvastatin reported in Table 1). Despite excluding these patients, there were no significant differences in rivaroxaban trough (46.2 ± 40.2 ng/ml vs. 44.7 ± 32.2 ng/ml; *t*‐test; *p* = .90) and peak (183.4 ± 112.8 ng/ml vs. 214.7 ± 109.8 ng/ml; *t*‐test; *p* = .37) anti‐Xa activity between atorvastatin‐treated patients and patients without atorvastatin.

Similarly, there were no significant differences in apixaban trough anti‐Xa activity between patients on atorvastatin and those without statin therapy (127.7 ± 71.1 ng/ml vs. 100.8 ± 61.1 ng/ml; *t*‐test; *p* = .12). Furthermore, the analysis of peak apixaban anti‐Xa activity did not reveal significant differences between those on atorvastatin and those without statin therapy (213.8 ± 103.6 ng/ml vs. 179.3 ± 72.9 ng/ml; *t*‐test; *p* = .14) (Figure 1). Finally, an analysis of apixaban anti‐Xa activity was performed excluding patients with amiodarone and verapamil co‐therapy. Even when patients who received amiodarone and verapamil were excluded, there were no significant differences in apixaban trough (126.6 ± 70.6 ng/ml vs. 98.9 ± 65.1 ng/ml; *t*‐test; *p* = .15) and peak (218.0 ± 100.7 ng/ml vs. 180.4 ± 77.1 ng/ml; *t*‐test; *p* = .13) anti‐Xa activity between atorvastatin‐treated patients and those not receiving atorvastatin therapy.

## 4 DISCUSSION

Atorvastatin, probably the most frequently used agent for the treatment of cardiovascular diseases, significantly reduces future cardiovascular mortality in the settings of the primary and secondary prevention of these diseases.^17^, ^18^ However, it could modulate drug metabolism, and therefore create an unexpected drug interaction with direct oral factor Xa inhibitors, as the metabolism of these directly acting anticoagulants is dependent on CYP and P‐gp activity.^1^, ^2^, ^3^ Several studies have previously demonstrated that atorvastatin could modulate CYP activity.^19^, ^20^ Furthermore, Rodrigues et al. showed in an experimental model that atorvastatin leads to decreased P‐gp function and modulates P‐gp synthesis in hepatocytes and peripheral blood mononuclear cells.^21^ Nevertheless, since no postmarketing studies have investigated the impact of atorvastatin on xabans anti‐Xa activity, we decided to do so. Although, the limitations listed below should be considered when interpreting the results of our study; the study did not show a significant interaction between atorvastatin and anti‐Xa activity in rivaroxaban and apixaban‐treated patients with AF. In addition, when patients with amiodarone and verapamil co‐administration were excluded from the analysis (due to the slight imbalance in this co‐therapy between patients treated with or without atorvastatin) there were still no significant differences in trough and peak anti‐Xa activity both for rivaroxaban and apixaban. A similar observation for rivaroxaban was previously reported from a phase I clinical study in healthy volunteers, where Kubitza et al.^22^ reported that there were no clinically relevant pharmacokinetic or pharmacodynamic interactions between rivaroxaban and atorvastatin. These data suggest that rivaroxaban can be co‐administered with atorvastatin without the risk of unexpected (too high or too low) anti‐Xa activity. Based on our data, this could also be applied to apixaban, as no significant impact of atorvastatin on trough or peak anti‐Xa activity of apixaban was noted. However, to the best of our knowledge, no other postmarketing studies for rivaroxaban or apixaban for the comparison of the results have been conducted (this includes other indications for direct oral factor Xa inhibition, such as venous thromboembolic disease). Therefore, the results of our observation should be confirmed in future larger studies.

The question of whether the results obtained in atorvastatin‐treated patients could be applied to all statin‐treated patients has also yet to be confirmed. Considering the fact that different statins differ in metabolism (and therefore, in possible pharmacologic interactions), it is possible that the risk of CYP‐related drug interaction would be lower in fluvastatin‐, pravastatin‐ and rosuvastatin‐treated patients, as these statins are not substrates of CYP 3A4, and higher in atorvastatin‐, simvastatin‐ , and lovastatin‐treated patients,^23^, ^24^ possibly preferring a “class” dependence in statin/direct oral factor Xa inhibitors interaction (as no significant interaction was seen with atorvastatin which has a higher probability of such interaction). Nevertheless, since the aim of our study was to explore the effect of atorvastatin, only atorvastatin‐treated AF patients were enrolled. Furthermore, to date, no other statins have been studied with regard to their possible interaction with direct oral factor Xa inhibitors metabolism. In summary, the question of whether a no relevant atorvastatin/direct oral factor Xa inhibition interaction could be considered as a “class effect” and applied to all other statins has yet to be confirmed and remains another issue which is open to future research.

### 4.1 Limitations

Our study had several limitations. First, the low sample size is probably the most important limitation. Second, the non‐randomized design is another limitation, and thus the data derived from our study do not have the evidentiary power of data derived from a randomized trial, and lack sufficient power to arrive at definitive conclusions. Nevertheless, due to robust data confirming the benefit of statin therapy for cardiovascular diseases, it would be difficult to randomize a patient who might benefit from statin therapy to receive a placebo. In addition, since our study was not designed to follow clinical end points, such as stroke, systemic embolism, and bleeding, clinical efficacy/safety data are missing. Therefore, the appropriate validation of our findings in a larger, prospective (randomized) study, with clinical end‐points is needed. Such study, enrolling atorvastatin‐treated AF patients and AF patients without atorvastatin on xabans, should follow these patients for the incidence of adverse ischemic (stroke or systemic embolism) and bleeding events. Moreover, anti‐Xa activity should be assessed and the correlation between this activity and the risk of adverse events would provide a final answer regarding the interaction between atorvastatin and direct oral factor Xa inhibitors. Third, in attempting to maintain the conditions of a “real‐world” clinical practice, we allowed an attending physician‐based decision on concomitant medication. This could lead to slight, statistically insignificant differences in possibly interacting medications, such as verapamil, or amiodarone in statin‐treated patients and those without statin therapy. These differences could in theory affect the results. Nevertheless, there were still no significant differences in xabans anti‐Xa activity when verapamil (5 patients) and amiodarone (12 patients) treated patients were excluded from the analysis. Therefore, this possible bias has a low probability. Furthermore, this study was designed to compare the effect of atorvastatin on direct oral factor Xa inhibitors activity, comparing this activity in atorvastatin‐treated patients and a control sample of patients without atorvastatin; it was not designed to compare the effect of different statins. Since we did not include patients with other statins (such as simvastatin, fluvastatin, or rosuvastatin) as another control sample, our results could only be applied to atorvastatin, and it is still not known whether other statins have any effect on the anti‐Xa activity of direct oral factor Xa inhibitors. In future studies, it would be interesting to extend the analysis to more widely used statins such as rosuvastatin. Finally, there is a lack of exact laboratory confirmation of drug compliance in our study (drug compliance was confirmed only by healthcare professional drug administration supervision).

## 5 CONCLUSION

This observational study did not show a significant impact of atorvastatin on trough and peak anti‐Xa activity in direct oral factor Xa inhibitors‐treated patients with AF, which suggests that atorvastatin could be safely co‐administrated with direct oral factor Xa inhibition. However, due to limited data and other limitations, further studies will be needed to adopt final conclusions.

## DISCLOSURES

Ingrid Škorňová, Matej Samoš, Tomáš Bolek, Lucia Stančiaková, Ľubica Vadelová, Peter Galajda, Ján Staško, Peter Kubisz, and Marián Mokáň declare that they have no conflicts of interest that might be relevant to the contents of this manuscript.

## AUTHORS’ CONTRIBUTION

I.Š., M. S, and T. B. designed the study, collected, analyzed, and interpreted the data and drafted the manuscript; I.Š. supervised anti‐Xa activity assessment; L. S. and Ľ. V. assessed the anti‐Xa activity, analyzed the data, and performed the literature search; P. G., J. S., P. K., and M. M. revised the manuscript critically. All the authors have read and approved the final version of the manuscript. All the authors agreed to be accountable for all aspects of the work in ensuring that questions related to the accuracy or integrity of any part of the work are appropriately investigated and resolved.

## ETHICAL APPROVAL AND INFORM CONSENT

This research was conducted according to ethical standards and approved by the local ethical committee (Jessenius Faculty of Medicine in Martin, Comenius University in Bratislava). The patients agreed to participate in the research and signed informed consents to their participation in the study.

## Data Availability

All data are available at Corresponding Author upon reasonable request.

## References

[1] Kvasnicka T , Malikova I , Zenahlikova Z , et al. Rivaroxaban ‐ metabolism, pharmacologic properties and drug interactions. Curr Drug Metab. 2017;18:636‐642. 28524005 10.2174/1389200218666170518165443

[2] Kubisz P , Stanciakova L , Dobrotova M , Samos M , Mokan M , Stasko J . Apixaban ‐ metabolism, pharmacologic properties and drug interactions. Curr Drug Metab. 2017;18:609‐621. 28440204 10.2174/1389200218666170424151551

[3] Guirguis E , Brown D , Grace Y , Patel D , Henningfield S . Establishing Edoxaban's role in anticoagulation. J Pharm Pract. 2016;29:228‐238. 27169733 10.1177/0897190016647314

[4] Testa S , Paoletti O , Legnani C , et al. Low drug levels and thrombotic complications in high‐risk atrial fibrillation patients treated with direct oral anticoagulants. J Thromb Haemost. 2018;16:842‐848. 29532628 10.1111/jth.14001

[5] Testa S , Legnani C , Antonucci E , et al. Coordinator of START2‐Register. Drug levels and bleeding complications in atrial fibrillation patients treated with direct oral anticoagulants. J Thromb Haemost. 2019;17:1064‐1072. 31013383 10.1111/jth.14457 PMC6852698

[6] Hirota T , Ieiri I . Drug‐drug interactions that interfere with statin metabolism. Expert Opin Drug Metab Toxicol. 2015;11:1435‐1447. 26058399 10.1517/17425255.2015.1056149

[7] Lennernäs H . Clinical pharmacokinetics of atorvastatin. Clin Pharmacokinet. 2003;42:1141‐1160. 14531725 10.2165/00003088-200342130-00005

[8] Holtzman CW , Wiggins BS , Spinler SA . Role of P‐glycoprotein in statin drug interactions. Pharmacotherapy. 2006;26:1601‐1607. 17064205 10.1592/phco.26.11.1601

[9] Steffel J , Verhamme P , Potpara TS , et al.; ESC Scientific Document Group . The European Heart Rhythm Association Practical Guide on the use of non‐vitamin K antagonist oral anticoagulants in patients with atrial fibrillation. Eur Heart J. 2018;2018(39):1330‐1393. 10.1093/eurheartj/ehy136 29562325

[10] Samoš M , Bolek T , Škorňová I , et al. Anti‐Xa activity in elderly Xabans‐treated patients with atrial fibrillation. Am J Ther. 2020;27:e507‐e509. 10.1097/MJT.0000000000001014 31356346

[11] Bolek T , Samoš M , Škorňová I , et al. Does proton pump inhibition change the on‐treatment anti‐Xa activity in xabans‐treated patients with atrial fibrillation? A pilot study. J Thromb Thrombolysis. 2019;47:140‐145. 30288664 10.1007/s11239-018-1748-5

[12] Samoš M , Bolek T , Stančiaková L , et al. Does type 2 diabetes affect the on‐treatment levels of direct oral anticoagulants in patients with atrial fibrillation? Diabetes Res Clin Pract. 2018;135:172‐177. 29175298 10.1016/j.diabres.2017.11.024

[13] Samoš M , Bolek T , Stančiaková L , et al. Anti‐Xa activity in oral factor Xa inhibitor‐treated patients with atrial fibrillation and a higher risk of bleeding: a pilot study. Blood Coagul Fibrinolysis. 2018;29:369‐373. 29538002 10.1097/MBC.0000000000000721

[14] Harding SD , Sharman JL , Faccenda E , et al. The IUPHAR/BPS Guide to PHARMACOLOGY in 2019: updates and expansion to encompass the new guide to IMMUNOPHARMACOLOGY. Nucleic Acids Res. 2018;46:D1091‐D1106. 29149325 10.1093/nar/gkx1121 PMC5753190

[15] Alexander SPH , Kelly E , Mathie A , et al.; CGTP Collaborators . THE CONCISE GUIDE TO PHARMACOLOGY 2019/20: introduction and other protein targets. Br J Pharmacol. 2019;176(Suppl 1):S1–S20. 31710719 10.1111/bph.14747 PMC6844537

[16] Alexander SPH , Kelly E , Mathie A , et al.; CGTP Collaborators . THE CONCISE GUIDE TO PHARMACOLOGY 2019/20: transporters. Br J Pharmacol. 2019;176(Suppl 1):S397‐S493. 31710713 10.1111/bph.14753 PMC6844579

[17] Nunes JP . Statins in primary prevention: impact on mortality. A meta‐analysis study. Minerva Cardioangiol. 2017;65:531‐538. 28249380 10.23736/S0026-4725.17.04323-7

[18] Schwartz GG , Fayyad R , Szarek M , DeMicco D , Olsson AG . Early, intensive statin treatment reduces ‘hard’ cardiovascular outcomes after acute coronary syndrome. Eur J Prev Cardiol. 2017;24:1294‐1296. 28504565 10.1177/2047487317708677

[19] Hukkanen J , Puurunen J , Hyötyläinen T , et al. The effect of atorvastatin treatment on serum oxysterol concentrations and cytochrome P450 3A4 activity. Br J Clin Pharmacol. 2015;80:473‐479. 26095142 10.1111/bcp.12701 PMC4574832

[20] Willrich MA , Rodrigues AC , Cerda A , et al. Effects of atorvastatin on CYP3A4 and CYP3A5 mRNA expression in mononuclear cells and CYP3A activity in hypercholeresterolemic patients. Clin Chim Acta. 2013;421:157‐163. 23501331 10.1016/j.cca.2013.03.007

[21] Rodrigues AC , Curi R , Britto LR , et al. Down‐regulation of ABCB1 transporter by atorvastatin in a human hepatoma cell line and in human peripheral blood mononuclear cells. Biochim Biophys Acta. 2006;1760:1866‐1873. 16996216 10.1016/j.bbagen.2006.08.003

[22] Kubitza D , Becka M , Roth A , Mueck W . Absence of clinically relevant interactions between rivaroxaban–an oral, direct Factor Xa inhibitor–and digoxin or atorvastatin in healthy subjects. J Int Med Res. 2012;40:1688‐1707. 23206451 10.1177/030006051204000508

[23] Schachter M . Chemical, pharmacokinetic and pharmacodynamic properties of statins: an update. Fundam Clin Pharmacol. 2005;19:117‐125. 15660968 10.1111/j.1472-8206.2004.00299.x

[24] Shitara Y , Sugiyama Y . Pharmacokinetic and pharmacodynamic alterations of 3‐hydroxy‐3‐methylglutaryl coenzyme A (HMG‐CoA) reductase inhibitors: drug‐drug interactions and interindividual differences in transporter and metabolic enzyme functions. Pharmacol Ther. 2006;112:71‐105. 16714062 10.1016/j.pharmthera.2006.03.003

